# Perfusion strategy and surgical repair of Type A interrupted aortic arch and Type 1 aortopulmonary window: a case report

**DOI:** 10.1051/ject/2025052

**Published:** 2026-03-13

**Authors:** Paula Leigh, Ed Harman, S. Adil Husain

**Affiliations:** 1 University of Utah Health, Primary Children’s Hospital, Division of Cardiothoracic Surgery Salt Lake City UT USA

**Keywords:** Interrupted Aortic Arch, Aortopulmonary Window, Pediatric Perfusion, Congenital Heart Surgery, IAA, AP Window, IAA with AP Window

## Abstract

*Overview*: Interrupted aortic arch in combination with an aortopulmonary window is an uncommon congenital cardiovascular defect. The anatomic separation between the aortic arch and descending aorta, combined with the communication between the ascending aorta and pulmonary trunk, leads us to a unique case where we can utilize the patient’s anatomic defect towards an efficient perfusion strategy. *Description*: The patient was a 19-day-old neonate who was initially discharged home after birth with no suspicion of congenital heart disease. The patient was later diagnosed with a type A aortic interruption with a large type 1 aortopulmonary window. Due to the unique combination of defects, surgery was accomplished utilizing single instead of dual aortic cannulation, and the repair was accomplished using a double patch technique. *Comment*: An individualized perfusion strategy was employed by taking advantage of the defect.

## Overview

**Figure 1:**
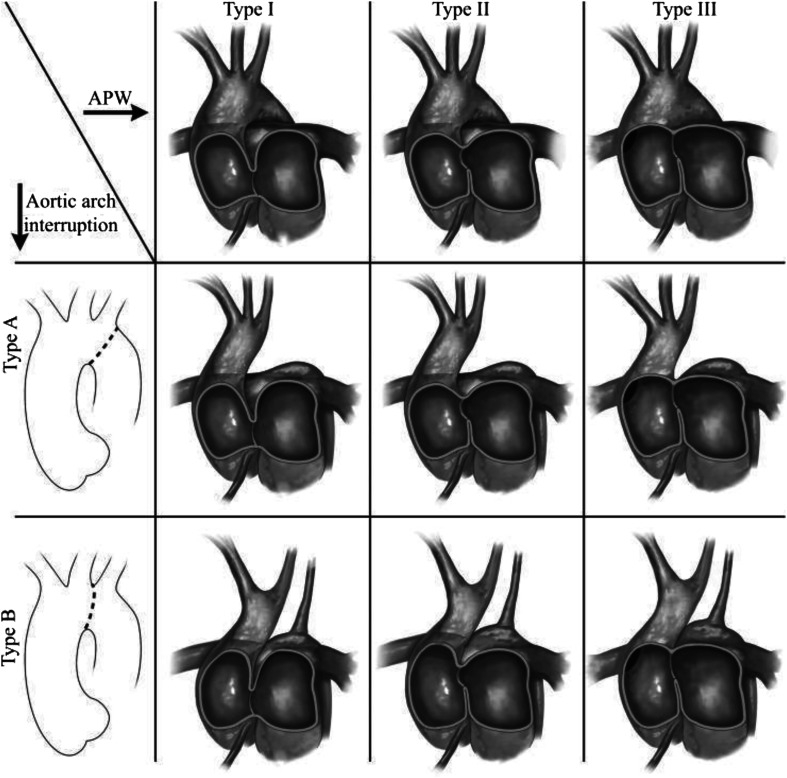
Co-occurrence of IAA with APW [4]. Permission to reproduce granted by the original author.

Interrupted aortic arch with aortopulmonary window is an extremely rare congenital cardiovascular defect. An interrupted aortic arch (IAA) makes up only 1.5% of all congenital cardiac patients [1]. This is an anatomical separation between the aortic arch and descending aorta and is classified secondary to the location of interruption. Distal perfusion is dependent on a patent ductus arteriosus, and thus patients receive a Prostaglandin E1 infusion following diagnosis [2]. The Celoria-Patton classification depicts the types of IAA based on the site of interruption. Type A interruption occurs just distal to the left subclavian artery, making up 30% to 40% of children with IAA [3]. Type B interruption occurs between the left carotid artery and the left subclavian (Figure 1) [4] and is the most common, making up approximately 53% of IAA cases [3]. Type C interruption occurs between the innominate and left carotid arteries and is the rarest, occurring in only 4% of IAA [3].

**Figure 2:**
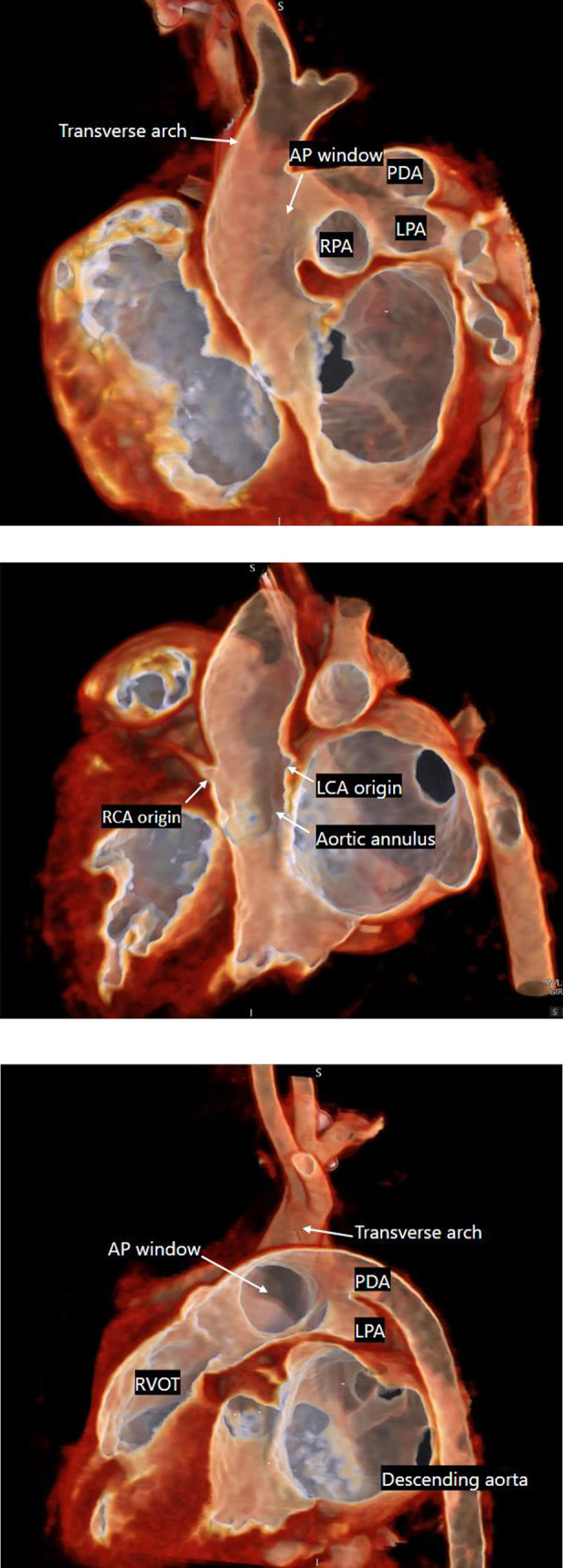
A. CTA image large APW in close proximity to transverse arch. B. CTA image identifying high origins of coronary arteries, above the level of the annulus. C. CTA image revealing distance between descending aorta and transverse aorta.

In conjunction with this condition, the prevalence of an aortopulmonary window (APW) makes for an exceedingly unusual combination. An APW is communication between the ascending aorta and the main pulmonary trunk. APW makes up less than 0.5% of congenital cardiac defects [5] and is associated 50% of the time with another defect [2]. The classification of APW has three categories based on the location of the defect. Type 1 is most common, and describes the proximal defect located just above the sinus of Valsalva [6]. Type 2 describes the distal defect located in the most superior portion of the ascending aorta. Type 3 describes the total defect involving the majority of the ascending aorta (Figure 1) [4].

IAA is commonly associated with a ventricular septal defect (VSD), where blood shunts from the left to the right ventricle. IAA is also commonly associated with DiGeorge syndrome, or 22q11.2 deletion [1]. In its rare form, IAA with APW shunts oxygenated blood from the ascending aorta across the AP window through a patent ductus arteriosus to the lower body, using the defect as a conduit from the ascending to the descending aorta. In combination with an IAA with APW, an atrial septal defect (ASD) is often present in 65% of cases [7]. With proper intervention, there is a very high chance of survival and a low chance of reoperation [7].

## Description

### Patient history

The patient was a term infant female born at an outside hospital (OSH) via c-section for breech presentation. No anomalies were noted upon physical exam, and the patient was discharged home with the mother on day of life (DOL) 3 and described as an “unremarkable normal newborn” in the hospital discharge summary. Patient presented to the emergency department (ED) at OSH on DOL 14 with chief complaint of shortness of breath. The patient was described as having rapid breathing and mild grunting starting two days prior to the ED visit, with low oxygen saturation. There was a differential between upper and lower extremity blood pressures and saturations. Chest X-ray revealed a mildly enlarged cardiac silhouette and increased pulmonary markings. These clinical and diagnostic findings indicated pulmonary overcirculation and potential for distal systemic malperfusion. The patient was started on prostaglandins (PGE) and transferred to our center with concern for undiagnosed congenital heart disease.

Upon arrival, an echocardiogram was performed, and the patient was diagnosed with type A interrupted aortic arch (IAA) and a large type 1 aortopulmonary window (APW). Additional findings were a large patent ductus arteriosus (PDA), a dilated left atrium (LA), an intact ventricular septum, and a small superior secundum ASD. The patient was admitted to the cardiac intensive care unit (CICU) and remained on PGE due to ductal-dependent systemic blood flow and supported with high flow nasal cannula (HFNC) therapy due to respiratory distress secondary to pulmonary overcirculation. A CTA was obtained which confirmed the diagnosis and revealed a large APW that nearly reached the transverse aortic arch towards the takeoff of the left subclavian artery (Figure 2A). Of note, the coronary arteries displayed origins above the level of the aortic valve commissural posts (Figure 2B). In addition, the distance of interruption appeared to be quite significant – nearly 2 cm between the ascending aorta and the native descending aortic tissue (Figure 2C). Surgical intervention was undertaken on DOL 19.

### Surgical and perfusion strategy

**Figure 3:**
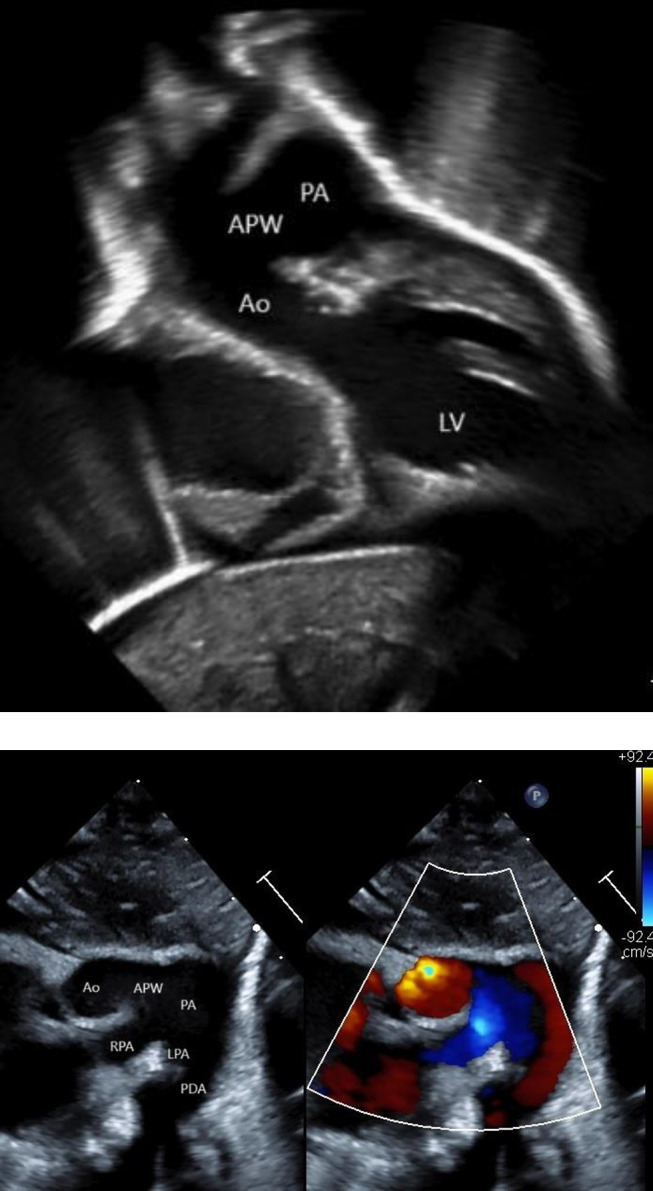
A. Echocardiogram identifying large APW. B. Echocardiogram illustrating flow through APW.

The patient’s formal preoperative diagnosis was IAA type A with a large PDA, APW involving the main pulmonary artery (MPA) and takeoff of the right pulmonary artery (RPA) (Figures 3A, 3B), and atrial septal defect. A cardiac index of 3.0 was used to determine full flows. She was 3.1 kg with a body surface area (BSA) of 0.21 m^2^, putting full flow at 0.63 L/min. An FX05 oxygenator and a Terumo neonatal 3/16 × 3/16 circuit were employed. Monitoring was accomplished using a right radial arterial pressure line and NIRS saturations on the cerebral left and right and somatic lower body left and right. Baseline MAP was 27 mmHg, and NIRS was 40 systemically.

**Figure 4:**
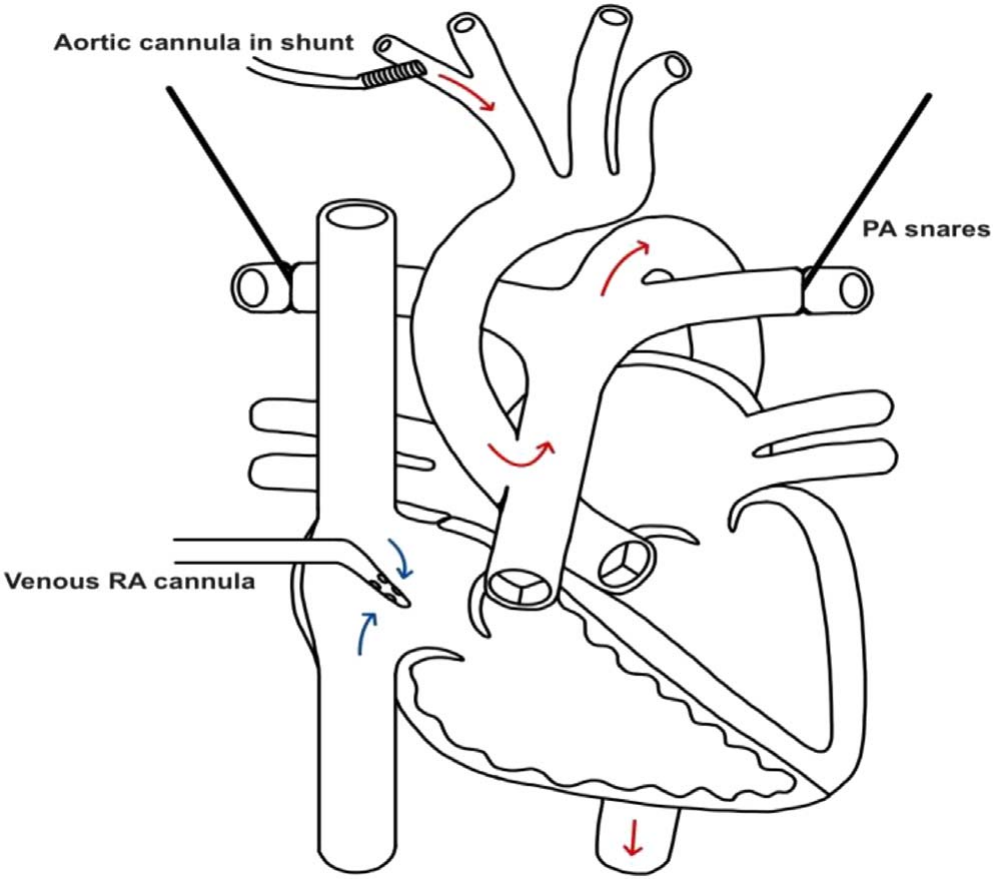
Flow pattern post cannulation through the defect.

Through a standard median sternotomy, the thymus was fully excised, and the pericardium was opened. The head vessels, large PDA, and branch pulmonary arteries were identified and isolated. Heparin was administered, and the patient was cannulated for bypass. A 3.5 mm Gore-Tex shunt was sewn to the innominate artery and cannulated with an 8 Fr aortic cannula. The right atrial appendage was cannulated with a 14 Fr cannula. Due to the large APW, a distal aortic cannula was not required for lower body perfusion (Figure 4). After adequate anticoagulation, cardiopulmonary bypass (CPB) was initiated. Target mean arterial pressure (MAP) on bypass was 35–45 mmHg. NIRS ranged from 70 to low 90 on bypass with an expected drop in somatic NIRS during antegrade cerebral perfusion (ACP), which was employed for arch reconstruction. During cooling and prior to the use of ACP, both branch pulmonary arteries were snared to direct flow through the APW into the PDA and to the descending aorta for lower body perfusion. A pH-stat CO_2_ strategy was utilized while cooling, and alpha-stat was reinstated when rewarming. After cooling to 20 °C, the PDA was ligated and divided, essentially placing the lower body on circulatory arrest, flowing at a 0.9 cardiac index (CI) to the entire upper body. At this point, ductal tissue was dissected from the thoracic aorta. A cross clamp was placed, and Del Nido cardioplegia was delivered antegrade into the aortic root at 20 mL/kg, which is the typical myocardial protective strategy at this institution. Clamps were placed on each head vessel, on the descending thoracic aorta, and each branch pulmonary artery, which redirected arterial flow up the innominate artery and right subclavian. ACP was initiated with a flow of 0.5 CI.

The large APW was opened, and the aortic tissue was divided from the MPA and RPA. The back wall of the native descending aorta was anastomosed to the native ascending aorta. The anterior arch was reconstructed with a large patch of pulmonary homograft material. The clamp on the thoracic aorta was removed, and the aorta was de-aired. Another cross clamp was placed, and a second dose of cardioplegia was delivered at 10 mL/kg at 53 min post XC. The snares on the head vessels were removed, and full flow CPB was reinstated with rewarming pursued. The right atrial cannula was directed into the superior vena cava. A right atriotomy was made, and a pump sucker-controlled the blood entering the right atrium from the inferior vena cava. The ASD was closed primarily. Following atriotomy closure, the venous cannula was redirected into the right atrium.

The MPA and RPA were reconstructed at the site of the APW using a large patch of pulmonary homograft material. With the left heart vented for deairing purposes, the patient was placed in the Trendelenburg position, and the cross clamp was removed. The heart regained normal sinus rhythm. After adequate rewarming, the patient was weaned from CPB and separated without issue. Modified ultrafiltration was performed, and protamine was subsequently delivered to achieve hemostasis.

Total CPB time was 156 min with an aortic cross-clamp time of 84 min. Upper extremity only flow time was 12 min with 44 min of low flow antegrade cerebral perfusion.

The patient returned to the Cardiac ICU with an open chest in stable condition. The patient’s lactate returned to normal, and on post op day 2, she underwent delayed sternal closure with no complications. On post op day 3, she was extubated and switched to a low-flow nasal cannula. The patient was discharged home on post op day 11.

### Comment

This patient’s rare congenital heart defect presented an opportunity for a unique perfusion strategy during surgical repair. In nearly all other IAA repairs, the surgical team must place a second arterial cannula in the PDA to provide lower body circulation. In the absence of APW, most patients also have a VSD, which will allow for mixing of oxygenated blood to the right side of the heart. In this case, however, there was an intact ventricular septum, and the APW provided mixing of blood from the left to the right side. As such, the APW was a direct conduit to the descending aorta through the PDA, allowing for the utilization of a single arterial cannula.

Despite having a rare congenital heart defect, patients diagnosed with IAA and APW often have a good prognosis and a high chance of survival with proper surgical intervention [3]. Without intervention, this cardiac malformation has significant morbidity and mortality. Both double-patch (employed here) and single-patch techniques have been shown to have good clinical outcomes with no difference in the success of the procedure. Single-stage repair is more common than staged repair for this defect [2]. Few patients require reoperations, and overall mortality remains low throughout childhood. The most common re-intervention for this defect is for aortic arch obstructions. Pulmonary stenosis may also occur, so the child should be followed as they grow and develop to ensure their great arteries remain competent [7]. For this patient, a single-stage repair with a double-patch technique was performed, which was necessary due to the limited amount of native tissue available for anastomosis and anatomical distance at the interruption between the ascending and descending aortas. Despite having a late diagnosis, our patient recovered quite well, showed no signs of permanent damage, and was discharged without concern.

## Data Availability

This was a case report, not a research paper.
